# Applying co-production principles in research: Reflections from young people and academics

**DOI:** 10.1177/09075682241269692

**Published:** 2024-08-10

**Authors:** Alice MacLachlan, Praveena Pemmasani, Isla Jamieson-Mackenzie, Christina McMellon, Emily Cunningham, Ruth Lewis, E. Kay M. Tisdall

**Affiliations:** 47970 University of Glasgow, UK; TRIUMPH Network Youth Advisory Group Member, UK; 47970 University of Glasgow, UK; University of Edinburgh, UK

**Keywords:** Collaboration, co-production, reflective practice, research, young people

## Abstract

Co-production, a form of collaborative working, is guided by principles including valuing all participants, building on individual strengths, blurring distinctions between roles, delivering benefits for all participants, building support networks and supporting people to deliver work themselves. This article explores how co-production is understood by young people and researchers and how co-production principles can be applied within a research context. We identify challenges of implementing existing co-production frameworks in research and key areas to consider for future work.

## Introduction

Co-production is a form of collaborative working first described by Ostrom and colleagues in the 1980s (Parks et al., 1981), which has become popular within policy, practice and research over the last two decades. Since it was first described, co-production has been used within a range of disciplines as a way of involving different stakeholder groups (Bandola-Gill et al., 2023; Masterson et al., 2022). In general, co-production is understood to involve bringing together the talents and experiences of all of those involved and sharing power and responsibilities in order to achieve outcomes not otherwise possible. However, the concept of co-production is still contested, with its purpose and definition varying across disciplines (Bandola-Gill et al., 2023; Ewert and Evers, 2014; Masterson et al., 2022).

Different rationales for undertaking co-production have been put forward (Martin, 2008; Williams et al., 2020). Technocratic rationales for co-production, refer to a model in which decision-makers are chosen based on their expertise in a specific domain, and focus on the potential for improved outcomes from collaborative working, particularly with subgroups who can provide ‘expert’ contributions. For example, a wide range of co-production literature focusses on service development in local government, healthcare and social care settings (Brandsen and Honingh, 2016; Masterson et al., 2022; Nabatchi et al., 2017) and the potential for co-production with service users to create more streamlined and efficient delivery (Nightingale et al., 2016; Sorrentino et al., 2018). In contrast, democratic rationales emphasise representation of the public in decision-making and the egalitarian benefits of co-production to marginalised groups (Fricker, 2007).

Guiding principles have been put forward to help facilitate meaningful co-production. Some of the earliest co-production principles were described in a series of papers developed in partnership between the New Economics Foundation (NEF) and Nesta, both third-sector organisations (Boyle et al., 2010, 2013; Boyle and Harris, 2009). These papers sought to bring clarity and structure to the field of public participation in public service development with a focus on the transformative potential of co-production. Boyle and colleagues outline six principles to facilitate meaningful co-production in service design (Boyle et al., 2010): recognising people as assets; building on people’s existing capabilities; mutuality and reciprocity; peer support networks; blurring distinctions; and facilitating rather than delivering (see Table 1 for a detailed description of each principle).

**Table 1.** Summary of young people’s and researcher’s reflections on the new economics foundation (NEF)/Nesta principles of co-production.

Principle	New economics foundation (NEF)/Nesta definition^a^	Summary of key points across both young people’s and researchers’ reflections
Recognising people as assets	Transforming the perception of people from passive recipients of services and burdens on the system into one where they are equal partners in designing and delivering services	- Importance of valuing all participants and identifying how they can contribute to a research project
		- Equal partnership may not always be appropriate, but there should be opportunities for meaningful involvement of young people at an appropriate level
Building on people’s existing capabilities	Altering the delivery model of public services from a deficit approach to one that provides opportunities to recognise and grow people’s capabilities and actively support them to put these to use with individuals and communities	- Supporting young people to be involved in research by building and developing existing and new skills
		- Offer a range of involvement opportunities that meet different individuals’ strengths and interests
Mutuality and reciprocity	Offering participants a range of incentives which enable people to work in reciprocal relationships with professionals and with each other, where they have mutual responsibilities and expectations	- Mutual benefits and expectations are important in developing co-production relationships and enabling young people to participate fully
		- Mutual responsibility may be inappropriate when co-producing research with young people
Peer support networks	Engaging peer and personal networks alongside professionals as the best way of transferring knowledge and supporting change	- Importance of relationships and peer support networks within the research team, particularly when working on sensitive subjects
Blurring distinctions	Blurring the distinction between professionals and recipients, and between producers and consumers of services, by reconfiguring the way services are developed and delivered	- Recognising young people’s lived experience as being of equal value to academic skills
		- Development of relationships over time supports the blurring of distinctions
		- Some distinctions are important to maintain (e.g., safeguarding roles) but can lead to power imbalances
Facilitating rather than delivering	Enabling public service agencies to become catalysts and facilitators of change rather than central providers of services themselves	- Supporting young people to participate in and produce research themselves, with opportunities across the whole research process
		- Importance of providing appropriate support to young people to facilitate their involvement
		- Potential tension between researchers and young people to deliver projects according to university and funder requirements

^a^Taken from Boyle et al., (2010), Public services inside out. Putting co-production into practice.

This article explores how co-production is understood by young people and researchers. The six principles proposed by Boyle et al. (Boyle et al., 2010) were used as a framework to guide discussion between young people, aged 16–25 years, and adult researchers involved in the Transdisciplinary Research for the Improvement of yoUth Mental Public Health research Network (TRIUMPH). Although Boyle et al.’s principles focus on service delivery rather than research, they offer an existing benchmark against which to measure the process of co-production (NESTA, 2012), and have been particularly popular in health policy circles. While there is a democratic rationale behind involving young people in decision-making processes that affect them (Montreuil et al., 2021), there are recognised challenges around co-production with young people including power imbalances and sharing responsibilities (Tisdall, 2017). We present a discussion reflecting on co-production within the research environment, including how existing principles can be applied and additional principles that may be relevant in this context.

TRIUMPH views collaborative writing with young people as a way to reflect the importance of co-production throughout the research process and to recognise the invaluable contributions that young people have made within the network. The writing team for the paper includes two young people from the TRIUMPH Youth Advisory Group (YAG; IJM, PP) and five academic researchers (EC; RL; AM; CM; KT) from TRIUMPH. The article’s outline and key discussion points were developed in online meetings with the full team and the lead author, AM, ensured that all team members were able to participate as fully as they would like to in shaping, drafting, and editing the article text. Rather than directly quoting excerpts from workshops with young people, the author YAG members independently wrote the paragraphs below on young people’s perspectives by integrating text provided by the wider YAG group during workshops into the article’s narrative, elaborating on points as necessary to support the discussion. By maximising the contributions made by the author YAG members, we ensured that the integrity of the text, i.e., the authentic voices and opinions of young people, was not lost to editing. This commitment to finding accessible ways to involve YAG members in the authorship team is in line with Miles’s et al. (2022) assertion that “incorporating collaborators’ perspectives via quotations misses an opportunity to attend to the co-produced nature of the knowledge conveyed in the publication, and paradoxically locates these collaborator(s) outside of the central ‘voice’ of the publication’s authorship”.

## Background: Co-producing research with children and young people

As co-production has become more popular in policy and services generally, it has also become more common, promoted, and even required within applied research (see Chief Public Health Officer, 2021; Durose et al., 2022). This fits with the move within childhood studies to encourage greater involvement of children and young people within research, not only as research participants but also in other influential roles, from research design to knowledge exchange (Collins et al., 2020). While often the terminology of co-production will be used to describe such research involvement, very similar activities are called by other terms such as co-design or participatory action research (Tisdall, 2021). Common across such research projects are commitments to share the power of knowledge creation, production, and exchange between adults, children and young people, who then work together throughout the research project (Redman et al., 2021).

A host of reasons are cited for co-producing research with children and young people. Methodologically, it may produce additional knowledge or even better research to contribute to the relevant field (Porter et al., 2012). Children and young people know the day-to-day of their worlds in ways that adult researchers cannot observe or extrapolate from their experiences (Brady et al., 2023). They may be better able to articulate the most relevant questions. For example, Cuevas-Parra and Tisdall (2019) report on child-led research that focused on bullying of Syrian refugee children, a topic not previously addressed by adult-led research projects. Additionally, children and young people can better engage their peers in primary research and design child-relevant fieldwork instruments by drawing from the shared experience of being a young person now, which adult researchers would find difficult to relate to (Ansell et al., 2012; Cunningham et al., 2024; Törrönen and Vornanen, 2014). Ethically, co-producing research is an important way to recognise and support children and young people’s agency, their competencies, and their participation rights (Dixon et al., 2019; Montreuil et al., 2021).

Children and young people regularly report on their positive experiences of co-produced research, both relationally in establishing trust, friendships, and community, and on their skills and knowledge development (Brady et al., 2023; Graham et al., 2016). There are several examples of how co-produced research projects have resulted in children and young people making a substantial and demonstrable influence on policy and practice, from young survivors of domestic abuse influencing legislative change in Scotland (Houghton et al., 2023) to children in Bangladesh improving rates of birth certification in their communities (Cuevas-Parra et al., 2019). Such research is proving more successful than many other participation activities with children and young people, where children and young people can be very frustrated when their views are not taken into account in policy or service decision-making (Tisdall, 2017, 2021). Thus, advocates for coproduced research with children and young people argue that it can make an important contribution to knowledge, respects and can enhance their agency, capabilities and skills, and in particular can be a powerful way for them to exercise their participation rights (as articulated under the UN Convention on the Rights of the Child).

The literature is rapidly accumulating on adult researchers’ reflections on coproducing research with children and young people, with some useful critique and subsequent discussion (e.g. Hammersley and Kim, 2021). However, there is far less on children and young people’s perspectives on its concepts, ethics, and enactment. This article makes a particular contribution on young people’s views on the very principles of co-production.

## Methods: Developing a conversation with young people about co-production

TRIUMPH is a research network that ran from 2018 to 2023, bringing together young people with academics, health practitioners, policymakers and those working with voluntary organisations to find new ways to improve youth mental health and wellbeing. TRIUMPH was funded by UK Research and Innovation (UKRI) as part of a large-scale investment in mental health research and capacity building across the UK. The core ethos of TRIUMPH was that young people should be involved in research about their own mental health. To achieve this, TRIUMPH established a YAG, a group of sixteen 16–25-year-olds recruited through four youth organisations from across the UK. During the four-year network, membership of the YAG evolved, but high engagement was maintained throughout, with some YAG members involved for the whole project period.

As part of the YAG, young people were involved in co-producing the network, including providing input on strategic and research funding decision-making, planning and delivering events, and leading their own research projects. The YAG’s activities were approved by the University of Glasgow College of Social Sciences Research Ethics Committee. YAG members were also involved in ongoing discussions about ethics, and both researchers and young people agreed upon the importance of maintaining the YAG as a space that enabled varied and challenging discussions, whilst respecting the dignity and confidentiality of members and the experiences they share.

Most YAG members were not familiar with the term ‘co-production’ when they joined TRIUMPH. Therefore, in order to co-produce network activities, it was important to establish a shared understanding of what co-production was and why it is important. This started a series of structured reflective discussions between YAG members and researchers about co-production that form the basis for this article. Researchers and young people took part in discussions as voluntary collaborators rather than research participants. Anonymised notes were made and recordings of meetings were anonymised at the time of transcribing. The only identifiable factor remaining in the data was whether comments were made by a young person or researcher.

In particular, this article draws on discussions from three workshops – two led by research staff and attended by YAG members (April 2021; April 2022), and the other led by a YAG member alongside a research staff member and attended by research staff members (March 2023).

An initial one-hour online workshop attended by eight YAG members was recorded via Mentimeter (https://www.mentimeter.com). In this workshop YAG members discussed their understanding and experiences of co-production within TRIUMPH. In a further 90-min face-to-face workshop, ten YAG members took part in a group discussion about the principles of co-production using post-its and large pads to capture key points, and then worked in pairs to write about the importance of each of the principles as well as two additional principles they had identified.

The third workshop, attended by five staff members, took place after the inception of this article and was intended to ensure equitable contributions and a well-rounded discussion. This one-hour online workshop covered each of the co-production principles in turn, asking staff to reflect on how these fit with their own experiences of co-producing research with young people. Research staff were also asked to comment on the additional principles identified by YAG members at the earlier workshops. Discussions were recorded, and key points noted using Padlet (https://www.padlet.com). These notes were used to develop the arguments presented in this article, with the meeting transcription used to add specific detail as required.

## Reflecting on the NEF/Nesta principles as a framework for co-producing research with young people

**Figure 1.**
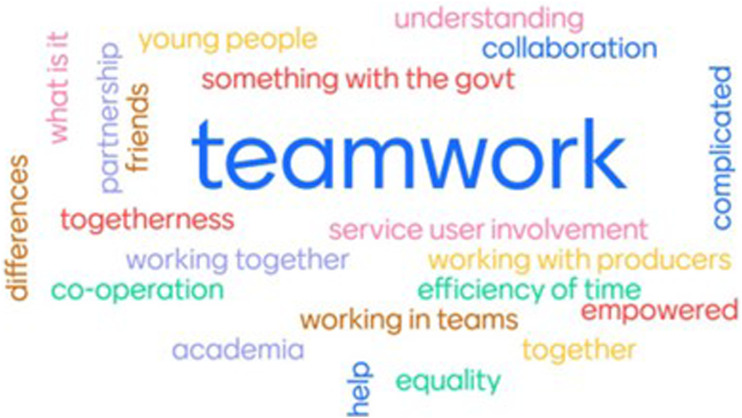
Word cloud developed from a workshop with TRIUMPH Youth Advisory Group members in April 2021 showing the first words that came to the minds of young people when they heard the word co-production.

For many young people in the TRIUMPH YAG, this was their first experience of co-production. Figure 1 shows the first words that came to the minds of YAG members when they heard the word *co-production*. Initially, the term *co-production* seemed vague and not distinct from young people’s previous experiences with collaborative working. However, the clear delineation of the co-production principles by NEF/Nesta clarified the concept and young people were readily able to understand its benefits and appreciated that TRIUMPH was built around these principles. During discussions YAG members were able to provide multiple examples of how each principle was met. However, young people also welcomed the opportunity to adapt Boyle et al.’s (2010) framework to suit the purpose of the project and the people involved. In the context of TRIUMPH, this meant (a) times when it was not feasible to strictly adhere to the framework, and (b) the introduction of additional principles.

Researchers involved in co-production for the first time as part of TRIUMPH reflected that the principles outlined by NEF/Nesta helped provide a framework to think about the way in which co-production should be done. However, for researchers more experienced in co-production with young people, the potential pitfalls in implementing the NEF/Nesta principles within research were more problematic. The wording of some of the principles and examples of how these have been implemented in service provision focus on improving service efficiency, and could potentially be seen as taking advantage of those involved when applied directly to a research context. As such, these principles may not lead to respectful and reciprocal working, with benefits for all those involved and not just the researchers. In particular, researchers commented on the considerable time and resource commitment required to set up and maintain effective co-production processes with young people (see also Brady et al., 2023). Rather than the view of co-production increasing the efficiency of service design and delivery, researchers remarked that co-production in research is often approached from a more value-based interpretation of increasing the impact and relevance of research and ensuring representation of marginalised groups, thus supporting both technocratic and democratic rationales put forward for co-production (Martin, 2008; Williams et al., 2020).

As well as these general reflections on the NEF/Nesta principles, young people and researchers discussed each principle individually. These reflections are presented below and the key points summarised in Table 1.

### Recognising people as assets

Boyle and colleagues define this principle in terms of viewing all participants as equal partners in designing and delivering services (Boyle et al., 2010), whereas young people took this principle to relate to valuing all participants and their contributions. Young people felt that this principle was the most important to them, and reflected that in order to value all participants the research process must be accessible, interesting to be involved in, and benefit those contributing their time and experience. Involving those from a wide range of ethnic, socioeconomic, and educational backgrounds, avoiding the use of academic jargon, providing training, and encouraging a transparent research protocol were seen as being important to make the research accessible to all. Interestingly, this understanding of the principle meant that contributions did not have to be equal but instead match participants’ ability and willingness. Young people recognised that all participants have limits and aimed to positively value their contributions, no matter how large or small.

Researchers thought that by framing participants as assets, this principle largely focussed on creating efficiency in service design and delivery, which may not translate appropriately into a research environment. However, the general focus of this principle on identifying what people can contribute to a project, rather than on what they cannot, was regarded positively. Researchers felt that it was important to have everyone around the table and ensure that all ideas were equally valued, whilst acknowledging the varying roles and responsibilities of different team members. This may mean that equal partnership is unrealistic. A key challenge faced by researchers was creating an equitable environment, in which young people were given the opportunity to contribute meaningfully to the research process, while being conscious that, unlike researchers, this was not their primary employment. Therefore, involvement opportunities and responsibilities needed to be provided at an appropriate level and may not be distributed equally between researchers and young people. Co-production, at least with children and young people, may not require *all* people to be involved at *all* stages of the research as the definitions often seem to mandate. As other authors have found (e.g. Brady et al., 2023), an inclusive process may instead support children and young people to be involved as they want to and are able to do, amongst their other responsibilities.

### Building on people’s existing capabilities

While Boyle and colleagues focus on building existing capabilities (Boyle et al., 2010), both young people and researchers understood this principle as supporting young people’s involvement in the areas of research they are most interested in. This meant recognising that young people are not trained researchers and therefore helping them build and develop skills to support their involvement.

Young people thought that the term *existing capabilities* presumed some degree of competence at research-specific skills, which can feel burdensome, especially if this is their initial or only exposure to research. Furthermore, young people may feel limited to taking up opportunities that utilise an existing skill set, rather than exploring their interests regardless of prior experience. Within TRIUMPH, YAG members appreciated that their capabilities were not presumed, and involvement was not restricted to their existing skillsets. Additionally, lived experience and research-adjacent skills (e.g., teamwork, communication, interpersonal skills) were viewed as existing capabilities, signalling to young people that they are qualified enough. Young people may also underestimate themselves or feel intimidated in an academic environment, leading to under-recognition and under-promotion of their abilities. Therefore, being supported by a project leader (one of the researchers) who can recognise strengths and weaknesses of team members and suggest appropriate roles/training was particularly valuable to young people. Liaising with a project leader to define the skills they need to carry out a task – and organising training where necessary – led to continued participation and maximum impact from their contributions. Training, which was directed by the young people’s needs, was provided informally by the researchers and, sometimes, other YAG members. However, the supportive role of a project leader can easily devolve into purely delegating tasks, where young people feel that the researchers ‘know best’, introducing a power dynamic.

From the researchers’ perspective within TRIUMPH, this principle involved researchers trying to provide an environment where young people felt comfortable to ‘give things a go’, even if it was something they had not done before. This approach required a certain level of trust from the young people that researchers would find appropriate ways for them to be involved in all aspects of the project. This was implemented with varying success, with feedback from young people indicating that some found the co-production process too fast while others found it too slow, highlighting the challenges of building new skills and finding ways to work in a group with a mix of previous experience and existing skills. In addition, researchers felt that there was often a push from funders to include young people who have not been involved in research before. This potentially discounted the benefits of involving those with previous experience in the deep and extensive participation necessitated by co-production. A nuanced consideration of how to ensure inclusivity, across different aspects of research, while also valuing skills and experience for certain roles and activities, was needed.

### Mutuality and reciprocity

Boyle and colleagues define this principle as providing a range of incentives that allow people to work in reciprocal relationships, with mutual responsibilities and expectations (Boyle et al., 2010). Both young people and researchers agreed that having mutual benefits and expectations helped establish co-production relationships, but that mutual responsibility for the delivery of the research was challenging when working with young people.

From the young peoples’ perspective, varied academic, financial and social incentives to be involved in research drove their involvement. Reflecting on their experiences, many YAG members initially joined TRIUMPH because of their interest in youth mental health and their wish to make a meaningful difference. Through their involvement, they experienced a range of other benefits, including building an array of skills, such as critical thinking and analysis, time-management, and interpersonal skills. Additionally, monetary incentives removed financial barriers to involvement by ensuring that young people could afford to participate (e.g., if they were missing work or other opportunities), thereby facilitating a more inclusive YAG with young people willing and able to be involved regardless of their background. Finally, YAG members acknowledged that though responsibility should also be mutual, it was less easily shared as, unlike the researchers, they were not employed on the project and therefore felt less pressure to meet expectations surrounding the delivery of the project. Instead, young people mostly saw the project as an extra-curricular activity, often secondary to pre-existing responsibilities such as education or employment.

Within TRIUMPH, expectations and responsibilities were discussed at the start of the project at a residential workshop and mutually agreed upon, ensuring that these were reasonable for young people. The main responsibility still lay with the research team for the delivery of the project as a whole, but areas where young people could be expected to contribute, and their anticipated level of involvement, were clearly defined. It was agreed that the main responsibility of the YAG was to ensure that network activities were relevant and accessible to young people. As such the YAG were expected to advise on a range of network activities such as funding decisions and event planning, as well as being involved in wider discussions around how the network should reach out to other young people. Expectations around behaviours and commitment levels were also put forward by the YAG, such as regularly attending meeting or providing apologies in advance, and responding to communications (usually via WhatsApp) in a timely manner. However, achieving the right balance of giving young people equal opportunities and responsibility to deliver on certain aspects of the project without overburdening them was a constant challenge for the researchers. In relation to the mutual benefits, researchers noted more ambitious ideas for TRIUMPH, better working practices and new research ideas evolving from the work with young people. As other teams have found (Liabo and Roberts, 2019), the researchers found the ethical and institutional requirements for compensation, reciprocity, and payment for children and young people tricky to navigate. While the older age of the young people involved in TRIUMPH made this easier than working with younger children, the solutions found still felt imbalanced in terms of power, recognition, and responsibilities.

### Peer support networks

Boyle and colleagues define this principle in terms of engaging peer networks as a way of transferring knowledge and supporting change beyond a specific service or organisation (Boyle et al., 2010). While this principle refers to participants engaging with external peer networks beyond the existing service or organisation, it is important to note that strong internal peer support networks within an organisation could be seen as being equally essential to successful co-production.

Within TRIUMPH young people’s external peer support networks were utilised to increase the reach of the network and helped to transfer knowledge and ensure it reached the people it pertained to. For example, YAG members used their external peer networks to share research messages and TRIUMPH activities (e.g., via social media accounts), to recruit for research studies, and to identify new YAG members interested in co-producing research. However, when it came to conceptualising this principle as part of a co-production approach, researchers and young people both felt this related less to the ability of peer networks to deliver outcomes and more about the relationship-building within and beyond the research team that allows for effective co-production. Young people felt that the relationships between themselves, supported by professionals, were the foundation of a productive research process and that these internal peer networks provided a crucial form of informal support for them. In the context of mental health research, after discussing potentially sensitive subjects, young people felt that they could turn to other YAG members to debrief in a more relaxed environment rather than seeking more ‘formal’ help from staff.

There was a significant focus on developing peer networks from the start of the TRIUMPH project. The young people involved in the YAG had different backgrounds and came from different locations across the UK, making the development of peer support within the group a primary focus before looking at the role of external networks. Though a time- and resource-heavy process, as other co-production teams found, building and supporting relationships is essential and not tangential (e.g. Collins et al., 2020; Pavarini et al., 2019). Both young people and researchers highlighted that providing spaces to build peer relationships and not rushing immediately into the development of research itself, particularly in the early stages of the network, reflected positively on the quality of the research. This was facilitated within TRIUMPH through residential workshops with the YAG and research team, particularly the inclusion of non-academic activities such as team dinners and escape rooms.

### Blurring distinctions

Boyle and colleagues describe this principle as blurring the distinction between professionals (researchers) and recipients (young people) by reconfiguring the way services are developed and delivered (Boyle et al., 2010). Young people felt that the core of blurring distinctions lay in recognising that their lived experiences are as valuable to the project as the academic skills that researchers bring to their role.

Within TRIUMPH, distinctions between the YAG members and the research team blurred progressively throughout the four-year project. For example, several young people moved between voluntary advisory member and contracted peer researcher roles during this period. Therefore, when young people moved from the ‘recipient’ YAG group to the ‘professional’ researcher group, their responsibilities shifted, and they shared greater responsibility for the delivery of specific aspects of the project associated with their role. Interestingly, despite being there in a similar professional capacity as the ‘adult’ researchers, peer researchers were not treated any differently by other YAG members. This is potentially because as relationships evolved over time, perceptions of distinction in professional versus recipient roles were broken down, even though the accompanying responsibilities were felt differently by individuals.

However, distinctions were not, and perhaps should not be, erased completely. Ostrom highlights that co-production relies on “individuals not “in” the same organization” (Ostrom, 1996). The YAG reflected that having roles and responsibilities distinct from those of researchers allowed them to maintain an exclusive identity within the co-production process. Conversely, researchers were mindful that they were young people who may require additional support and safeguarding. Ultimately, researchers were responsible for research delivery and young people’s well-being, creating tension as some power dynamics always remained in place. The responsibility of the researchers to safeguard competes with the co-production objective of treating young people as equal participants with full agency, i.e., young people that bear the full responsibility for their actions.

### Facilitating rather than delivering

Both young people and researchers readily understood how this principle translated into the research context. Young people understood this principle to be about supporting people and enabling them to participate in and produce research themselves rather than it solely being the researchers’ task. They wanted to see opportunities for young people to contribute to academic research through involvement in study design and the subsequent write-up, and not be confined to the data collection or discussion aspects of a research project. In this way, young people can better help to refine research to target the most current and urgent needs of themselves and other children and young people. However, young people highlighted the importance of having support from trained researchers to equip them with the necessary information and skills to play an active role in research. In addition, young people did not necessarily feel the need to all be involved in all aspects of a project. Some instances were highlighted where young people preferred to step back from certain roles, e.g. when they were not interested in the tasks or had other commitments, meaning that researchers took on a greater role. This particularly occurred when deadlines were nearing and researchers found it more difficult to be flexible and facilitate while delivering project outputs in a timely manner.

Within TRIUMPH, YAG members had the opportunity to conduct their own research project, for which the young people came up with the idea and research design, carried out data collection, and analysed and wrote up the project findings. The ability to support young people to deliver their own research was possible as TRIUMPH allowed flexibility around funding research projects and provided a significant amount of staff time and resources to facilitate this project. However, as reported by others (Flinders et al., 2016; Oliver et al., 2019), researchers recognised that in many other cases they had to abide by the guidelines, rules, and procedures set by the university/funder, and therefore being able to co-produce research using this model can be more challenging. For example, the requirement to define all project activities and outputs when applying for funding can make it difficult to then modify plans as a result of the co-production process once the project has started. However, TRIUMPH was unique in that resources were specifically set aside for the young people’s project without needing to define what the research might look like in advance, and could be used to develop creative outputs rather than academic papers if they wished.

## Key challenges to implementing co-production principles with young people

Although the NEF/Nesta principles provide a useful framework for approaching co-production, three key challenges were identified to implementing these six principles as originally described.

### Time and resource commitments

While Boyle and colleagues focus heavily on how co-production can lead to increased efficiency and streamlined service development, within research, co-production aims to increase the relevance and impact of the research within target groups. For example, the principles of building on existing capabilities – understood to also include developing new skills among participants – and peer support networks – largely focussing on building strong relationships – require extensive time and resource commitments and are, therefore, unlikely to be seen as efficiency savings in research. However, this concept of increasing efficiency is a by-product of forming co-production principles centred around service development. Instead, we propose that efficiency should be secondary to the value that co-production in research lends to its stakeholders and findings.

### Equality

Equality issues were raised in terms of expectations around contributions and responsibility for the work. Both young people and researchers highlighted that effective co-production should not mean equal sharing of work; instead, everyone should be equally involved in decision-making and be offered equal opportunity for further contributions. The idea of ‘pockets of participation’ provides participants ownership of specific areas of a project where young people may contribute equally or take equal responsibility for delivering specific aspects of a project but does not require them to shoulder inappropriate responsibilities (Franks, 2011). This may be a more feasible approach to involving young people in research, as it provides areas where it is more appropriate for researchers to facilitate rather than deliver and for distinctions between roles to be blurred, rather than aiming for complete equality between young people and researchers across a whole research project. For example, the TRIUMPH YAG were heavily involved and shared responsibility for leading their own youth-led research project and for delivering a two-day event aimed at young people, researchers, and practitioners. In contrast, they were less involved in other aspects of the network such as regular stakeholder communications and engagement, and project reporting.

### Power Dynamics

Though the principles of blurring distinctions and mutuality and reciprocity advise equal sharing of power amongst all participants for successful co-production, we identified issues around power dynamics in the relationships between young people and researchers. Unequal power in the relationships between young people and adults often exist as a result of differences in age, status, competency, and experience (El Gemayel and Salema, 2023). While the authors agree that young people should be empowered to take part in research and contribute their ideas and lived experiences, it was felt that it was appropriate for researchers to continue to take overall responsibility for the delivery of a project and be accountable to funders and academic organisations. However, this made it difficult to share power equally in all circumstances as researchers sometimes had to make decisions to meet the project obligations.

## Additional principles to support co-production of research with young people

During discussions young people and researchers identified concepts, additional to existing NEF/Nesta principles, which they felt needed consideration when co-producing research with young people.

### Flexibility

The first additional principle, identified by young people, was the willingness of researchers and participants to be flexible and adaptable. Young people wanted the research to adapt to their changing circumstances, feelings, and abilities and thought this could be done through continuous feedback and reflection. Examples of flexibility appreciated by the YAG during the TRIUMPH project included: the option to keep the camera off and use the chat function instead of speaking during virtual meetings; regular downtime during residentials; and being able to feedback on sessions verbally, written down, or via an online survey. For the researchers, this flexibility extended to communication methods (e.g., using WhatsApp over email) and working hours, often scheduling evening meetings to accommodate young people’s work/education commitments. However, researchers highlighted the pressures of ‘getting things done’ whilst remaining flexible and felt that these responsibilities were not always considered a priority by young people. Nevertheless, flexibility in co-production is likely to take different forms depending on the specific needs of those involved, which should be raised early on and regularly reviewed to make suitable adjustments.

### Respecting boundaries

A second principle identified by young people was respecting boundaries, in order to create a constructive and accessible environment where they could contribute fully to the research. Each young person and researcher may have specific topics or activities they find uncomfortable or inaccessible. To safeguard everyone involved and protect the integrity of the co-production process, these boundaries must be recognised and respected. Specific examples of boundaries identified by the YAG included: establishing and respecting working hours; acknowledging that some have certain off-limits topics and not expecting an explanation if they do not wish to take part; and respecting that not every young person or researcher will be comfortable with certain humour.

### Honesty

A final additional principle, identified by researchers, was honesty. While this links to mutuality and reciprocity and blurring distinctions, researchers felt that the ability to have honest and transparent discussions with young people, e.g., about the constraints of the research process such as funding and time and more personally about researchers’ vulnerabilities, was a key part of working with young people as partners in research.

Overall, these additional principles should be considered in any co-produced project. However, the principles of respecting boundaries and honesty are particularly pertinent to working with young people, where power imbalances are more likely to persist.

## Co-production in context

The particular context within TRIUMPH has contributed to the way the young people involved in these discussions experienced co-production. Unlike short-term research projects, TRIUMPH involved young people in the project over an extended period of 4 years – a significant time in a young person’s life, during which they will change personally, professionally, and socially, and evolve in terms of their interests and experiences. This presented both challenges and advantages. There was some turnover of young people, resulting in the need to continually adapt working practices and relationships to incorporate the needs of new members. However, the long-term nature meant that as young people became more embedded in the project, they contributed more to the project and voiced novel and exciting ideas for the research going forward, in a way that the more common shorter, light-touch engagement may not have facilitated. This made it easier to implement some principles, such as blurring distinctions between roles and facilitating rather than delivering the project.

The COVID-19 pandemic also transformed the way researchers worked with young people. Originally, TRIUMPH had planned to work the YAG in a more structured way, with a focus on involving wider groups of young people in the network. However, building external networks proved challenging with the pandemic, shifting the focus to how YAG members themselves could contribute to research. This provided an opportunity to implement the principles of co-production differently while working more closely with a focussed group of young people. During the pandemic, alongside structured online meetings to develop research projects, young people and researchers chose to attend virtual socials to stay connected, which strengthened relationships and contributed to the development of strong peer support networks. The change in approach to online working also supported researchers to implement the principle around recognising people as assets, as young people were able to join meetings and events more easily, without the need to travel, and so were able to contribute to decision-making and delivery across a wider range of project activities.

This article focuses on the principles of co-production defined by Boyle and colleagues for NEF/Nesta as this was the most relevant framework available at the time of the inception of TRIUMPH in 2017/2018. Since TRIUMPH commenced, co-production guidance and frameworks specific to research have proliferated (Campbell and Vanderhoven, 2016; UK Research and Innovation; NIHR, 2024). Most notably, the National Institute for Health and Care Research (NIHR) has published guidance on co-producing a research project that defines five key principles: sharing of power; including all perspectives and skills; respecting and valuing the knowledge of all those working on the research; reciprocity; and building and maintaining relationships (NIHR, 2021). These principles were developed to strengthen public involvement in research (Staniszewska et al., 2018), based on co-production principles previously derived for service development such as those put forward by NEF/Nesta (Boyle et al., 2010; Boyle and Harris, 2009). The NIHR principles align more closely with young people’s and researchers’ interpretations of the NEF/Nesta principles in several ways including: an emphasis on relationships; ensuring all views are included and treated equally even if equal contribution is not possible; and making sure everyone involved benefits from working together. However, issues around the sharing of power and responsibility remain, particularly when working with young people. While not explicitly including principles around flexibility, boundaries, and honesty, the NIHR guidance partially covers these additional points by highlighting key features that one might expect in a co-produced research project such as establishing ground rules, having an ongoing dialogue, and flexibility within the research process (NIHR, 2021).

## Conclusions

This article provides a critique of an existing co-production framework based on the experiences of researchers and young people involved in the TRIUMPH Network. We highlight that co-production approaches such as those originally presented in frameworks like the NEF/Nesta principles need to be adapted to different contexts and populations in order to meet specific project needs. We have highlighted specific challenges around the involvement of young people in the co-production of research, such as power imbalances and equality, and also identified that successful co-production requires relationships to be central to the way research is conducted, and for research systems to flexibly adapt goals and address young people’s needs. As experiences of co-production proliferate, all those involved in co-production should continue to critique and develop practices and frameworks to best support this type of research. Collaborative reflection on the theory, practice and ethics of co-production can help ensure that the principles of co-production are borne out in the process of refining what co-production is – and what it is not – within the research context, thus leading to more purposeful discussion around how to improve it.
